# Charles Henry Turner and the cognitive behavior of bees

**DOI:** 10.1007/s13592-021-00855-9

**Published:** 2021-04-08

**Authors:** Martin Giurfa, Anaclara Giurfa de Brito, Tiziana Giurfa de Brito, Maria Gabriela de Brito Sanchez

**Affiliations:** 1 College of Animal Science (College of Bee Science), Fujian Agriculture and Forestry University, Fuzhou, 350002 China; 2 Centre de Recherches sur la Cognition Animale, Centre de Biologie Intégrative (CBI), University of Toulouse, CNRS, UPS, 31062 Toulouse cedex 9, France; 3 Institut Universitaire de France (IUF), Paris, France; 4 UCL Anthropology, University College London, London, WC1H 0BW UK; 5 Sciences Po Toulouse, CS 88 526, 31685, cedex 6, Toulouse, France

**Keywords:** Charles Henry Turner, Black Lives Matter, social bees, solitary bees, cognition

## Abstract

Social movements in several countries are stimulating a reconsideration of academic structures and historic figures and promoting reparation and recognition of marginalized and forgotten black scientists. A paradigmatic case in that sense is Charles Henry Turner (1867–1923) who was the first African American to receive a graduate degree at the University of Cincinnati and one of the first in earning a PhD degree of the University of Chicago. He performed numerous experiments on sensory perception, orientation, and mating of solitary and social bees, most of which have been unjustly forgotten despite the fact that they anticipated fundamental concepts of animal cognition. We review these studies and highlight the importance of his ideas for modern views of animal cognition and the study of bee behavior. We conclude that besides his scientific contributions, Turner is an inspiration for scientists fighting against social adversity and prejudices.

Since the start of political actions occurring in several countries following the death of George Floyd at the hands of Minneapolis police, the movement “Black Lives Matter” has gone mainstream and is the cause for a deep questioning of heroes, myths, and the way history and national identity have been built over decades. This attitude has reached academia where inequalities and social injustices may occur, reflecting the social structures in which academic institutions are embedded.

Within this framework, vindicating merits and findings by black scientists unfairly forgotten is necessary for constructing unbiased scientific knowledge. Apidologists, and more generally speaking, scholars interested in entomology and animal behavior, have the opportunity to achieve this reparation by acknowledging the work of Charles Henry Turner (1867–1923), an African American scientist who produced numerous contributions in the fields of entomology, animal behavior, and general physiology, among others (Figure 1) (Abramson 2009).

**Figure 1.**
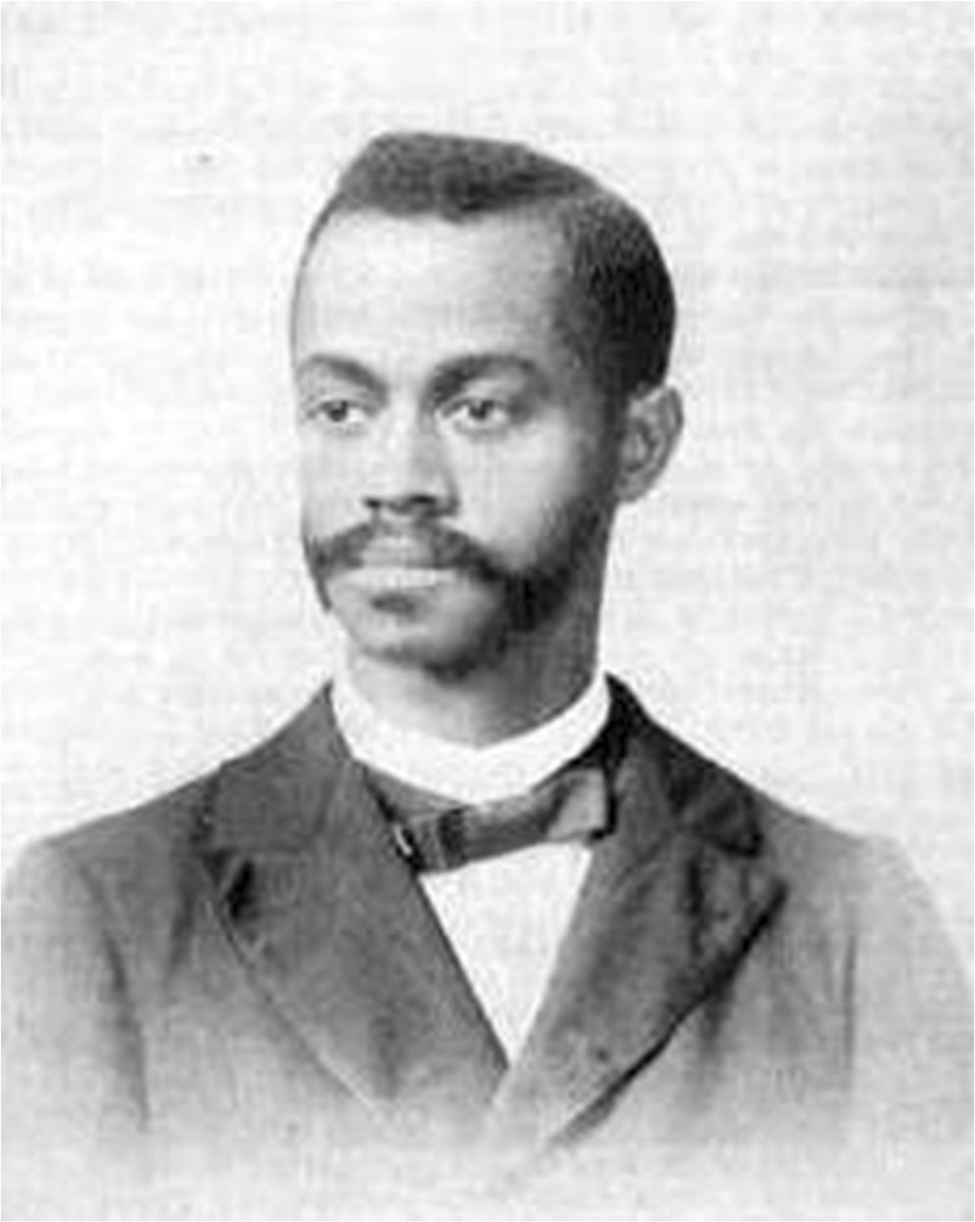
Charles Henry Turner. From Encyclopedia Britannica (public domain).

Turner was born in Cincinnati, Ohio, in 1867, and studied biology at the University of Cincinnati. After earning his B.S. degree in 1892, and being the first African American to receive a graduate degree from this University, he published several studies (including a synthesis of his B.S. thesis in Science (Turner 1892a), followed by another publication in the same year in the same journal (Turner 1892b)). Despite this remarkable success, all his attempts to obtain a position in academic institutions were unsuccessful. He managed, nevertheless, to earn a PhD in Zoology from the University of Chicago in 1907, being probably the first African American receiving a PhD degree from this institution. He then applied for a position at this university, but was again rejected due to the dominant racism that impregnated academic spheres. As he was already married and had two sons (one named Darwin Romanes Turner, showing his profound adherence to Darwin’s evolutionary views), he had no choice but to look for a position outside academia. One could easily imagine the frustration of the young Turner, with a family in charge, with several papers published, two of which in *Science*, getting, one after the other, successive rejections of several academic institutions based on racial issues that impregnated all levels of the society in which he had to live. In 1908, he was finally appointed as a teacher in a high school for African Americans, the Sumner High School in St. Louis, where he stayed until his retirement in 1922. Bibliographical accounts on Turner mention that he received an inappropriate pay and had a heavy teaching load at that school, and that this may have caused the myocarditis that killed him in 1923, at age 56 (Du Bois 1929; Abramson 2009). His death certificate indicated wrongly that his occupation was “druggist,” perpetuating thereby the injustice he faced during his life as a scientist (Abramson 2009).

Remarkably, and despite the recurrent frustrations he experienced throughout his life, he was able to perform dozens of experiments in the fields of animal behavior and entomology, producing important contributions that anticipated modern visions and concepts to various extents. He published 71 papers and made fundamental discoveries on animal behavior. Some of them were on social insects, particularly bees, wasps, and ants, which were some of his favorite and most investigated animals.

## Turner’s scientific contributions

Summarizing here Turner’s scientific contributions is difficult given the extent and diversity of his numerous works (for an extensive review, see Abramson 2009; Dona and Chittka 2020). He addressed topics such as comparative neuroanatomy in both vertebrates and invertebrates, arthropod taxonomy, insect behavior—with a particular focus on insect navigation—insect learning, spider behavior, audition in moths, leaf morphology in grapevines, and even civil rights. His neuroanatomical accounts of the avian (Turner 1891, 1892a) and invertebrate brain (Turner 1899) emphasized his evolutionary views.

Many of his studies focused on the behavior of social insects as he was deeply attracted by their social organization, division of labor, and collective intelligence. This occurred at a time in which dominant science tended to view and describe insects as rudimentary creatures with limited senses and capacities. Terms such as “taxis” (the innate movement of an organism towards or against a stimulus such as light) and “tropism” (the orientation—without necessarily a movement—towards or against a stimulus) (Franck 1985) were commonly used to describe entirely the behavior of insects. In other words, insects (and social insects among them) were viewed as primitive creatures, reacting only instinctively to external stimuli, without any other remarkable capacity.

Turner, a careful observer of social insects in their natural context, rejected this preconception. A leading idea in many of his works was that insects do not behave purely based on taxia or tropisms but that they exhibit “intelligent behavior,” which he tried to analyze using different experimental paradigms for studying problem solving (e.g., Turner 1913). In this way, he pioneered, without being necessarily credited for that, current cognitive views on insect behavior, which emerged many years later (Griffin 2001). Indeed, thanks to intensive research performed since the nineties, which introduced a “cognitive revolution” in the field of studies on insect behavior, we have understood that insects, in particular Hymenoptera, are endowed with remarkable learning and memory capacities, going well beyond simple associative learning (Giurfa 2003). Bees, for instance, categorize visual images (Benard et al. 2006), learn to solve problems based on concepts (Avarguès-Weber and Giurfa 2013), have a sense of number, and can even perform basic addition and subtraction (Giurfa 2019).

Yet, this was not the established view in Turner’s time. Against the existing mainstream, Turner provided accurate descriptions and analyses of the behavior exhibited by bees, ants, wasps, and caterpillars (Turner 1907a, b, 1908a, b, 1918), and proposed that memory was a fundamental property of the navigation strategies employed by these insects. His conclusions anticipated by several decades the cognitive perspectives adopted at the end of the 1990s to characterize insect behavior (Menzel and Giurfa 2001; Giurfa 2003). He concluded, for instance, that “ants are much more than mere reflex machines; they are self-acting creatures guided by memories of past individual (ontogenetic) experience” (Turner 1907b).

These achievements contrast with the treatment he received from academic institutions (Du Bois 1929; Abramson 2009). Precisely, his works are particularly remarkable because they were done in such adverse conditions: Turner had no access to institutional laboratory resources or libraries, as well as undergraduate or graduate students, and performed most of his work from the disadvantaged position (compared to scientists established in academic institutions) of a high school teacher.

## Turner and the solitary bees

Turner was a passionate observer of bee behavior. He wrote contributions on solitary bees, parasitic bees, and, of course, honey bees.

**Figure 2.**
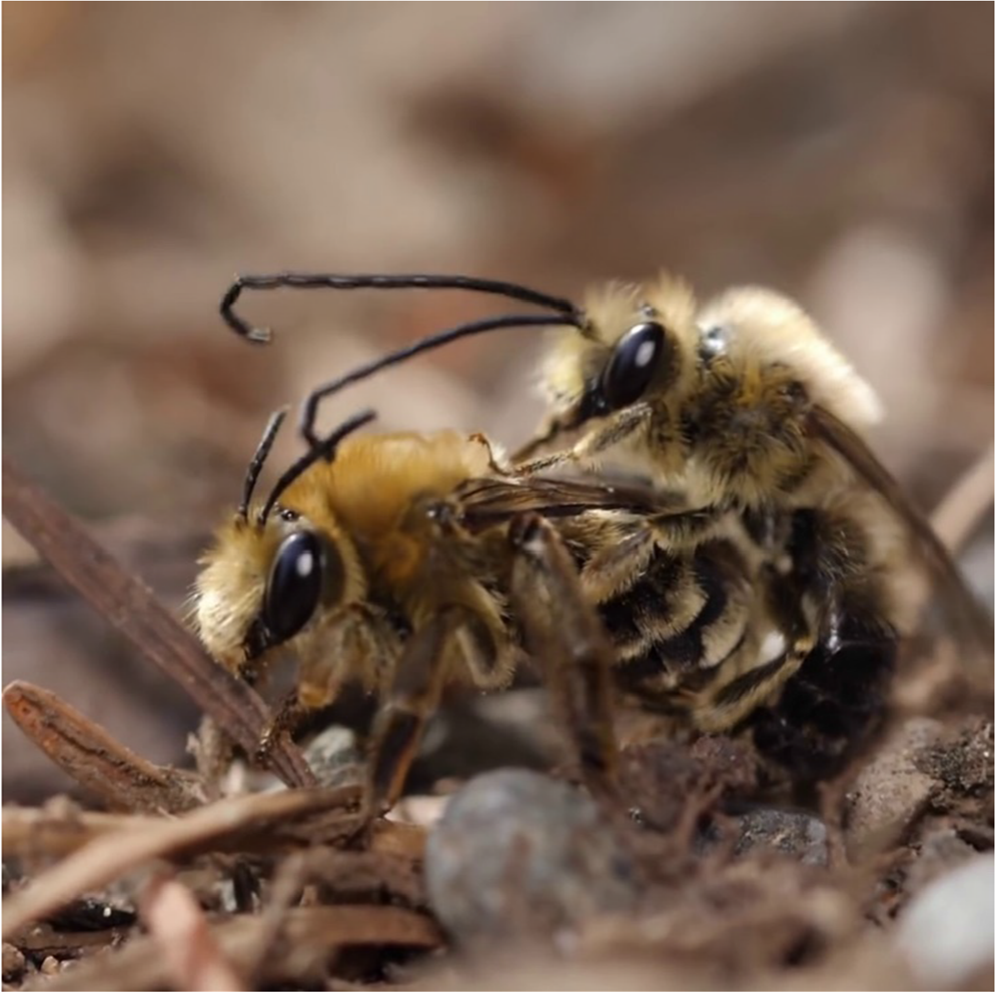
The mating of *Melissodes* bees. Courtesy of Karla Thompson @Karlaii.

He described for the first time the nuptial flight of long-horned Bees *Melissodes* sp. (e.g., *Melissodes communis*), a solitary bee species with a generalist diet that ranges across the eastern and southern US and that nests in burrows on the ground (Figure 2). Females emerge in mid- to late September and provision burrows with pollen until the first cold days of October or early November. Mating occurs in the nesting area, with males chasing females in complex flight maneuvers, grasping them either in flight or at the ground level and rolling with them repeated times in what Turner described as a “dance” in an article full of poetic impressions (Turner 1908c): “No skilled musician plays entrancing tunes, but as they dance, each bee makes music with its wings.” After describing this complex parade (Figure 2), Turner concludes that this behavior is a “nuptial ambuscade since it is a device, which promotes sexual union.” This could be obvious today, but at a time, in which this behavior had neither been described, nor analyzed, the eye of a scientist was required to make the correct conclusion.

Another important work with historic implications (despite the fact that it remained ignored by scientists working in insect navigation) is Turner’s study on the homing and spatial orientation of these *Melissodes* bees (Turner 1908a). Again, it is important to place the study in its appropriate historical context, even if such framework appears outdated today. The dominant idea prevailing at Turner’s time—and this is precisely the starting point of his article—was that the flights of insects were mainly guided by anemotropism (orientation with respect to the wind direction) and phototropism (orientation with respect to the sunlight). This appeared insufficient to Turner, who spent hours observing the flight behavior of *Melissodes* bees around their nest burrows.

Turner performed experiments in the field after finding an abandoned garden in which these bees had established their nests. He recorded the departing and arrival direction and orientation flights of bees after adding or suppressing prominent landmarks around their burrow (e.g., a tent of white paper). He even dug similar burrows close to the real nest and displaced the landmark close to the dummy burrows to determine their influence on the bees’ choice. His results were conclusive, yet ignored for several years: the bees learned the nest location relative to that of the surrounding landmarks so that when these were displaced, the bees searched at the wrong location, but at the position at which the burrow would be expected relative to the landmark. Turner’s results thus showed the presence of associative learning (bees learning to associate the nest with specific visual cues provided by landmarks) and of visual memories as this information was used after returning to the nest. The search behavior of bees could only be explained if the animal retrieved from a memory store the information that guided its decision. Turner concludes, “By a process of elimination, the most consistent explanation of the above behavior is the assumption that burrowing bees utilize memory in finding the way home, and that they examine carefully the neighborhood of the nest for the purpose of forming pictures of the topographical environment of the burrow.” He even specified that the process of forming these memory pictures occurs upon departure from the nest, in particular if modifications of the surrounding landmarks were introduced experimentally.

These experiments remind a classic work performed by the Nobel Prize winner Niko Tinbergen two decades after Turner’s observations. Tinbergen studied the orientation behavior of the digger wasp *Philanthus triangulum,* which also nests in a burrow on the ground (Tinbergen 1932; Tinbergen and Kruyt 1938). He found that wasps localized their burrow using a constellation of surrounding visual landmarks within a radius of 200 cm. Like Turner, he placed artificial landmarks (e.g., pine cones) around the nest, and he either modified their spatial configuration (e.g., triangle vs. circle) or displaced them to new locations. The same result was found: the wasps were sensitive to the location and configuration of landmarks, and used this information to guide their return to the nest. Yet, for Tinbergen, the question of the mechanism underlying the wasp’s performance was not so important. He discussed the orientation behavior of the wasps using Kühn’s taxis nomenclature (Kühn 1919) and classified his own observations as a case of “mnemotaxis,” a taxis guided by a sort of stored memory (Tinbergen and Kruyt 1938). Yet, he also realized that the wasp behavior did not follow the reflexive assumptions of Kühn’s mnemotactic hypothesis as the wasps approached the landmark constellations from different directions. The discussion of the mnemotaxis idea appears only at the end of his article and was described as “a formal gesture” to adjust to an existing nomenclature rather than a thorough reflection on the underlying mechanisms of orientation (Roell 2000). Turner, on the contrary, was briefer but more direct. He did not hesitate to use the terms “learning” and “memory” to describe the bees’ orientation behavior.

Turner’s ideas on the memorization of visual pictures around the nest reappeared 75 years later to account for honey bee navigation and close-up orientation. British scientists performing experiments on landmark learning by honey bees proposed the idea that bees take “visual snapshots” of the hive and food source surroundings and that their choice is guided by a comparisons between currently perceived images and memorized snapshots (also called “eidetic images”). High overlap between both indicates appropriate orientation and identification of the goal (Cartwright and Collett 1982, 1983). Yet, in between, Turner’s ideas had been lost and his work was again not cited.

**Figure 3.**
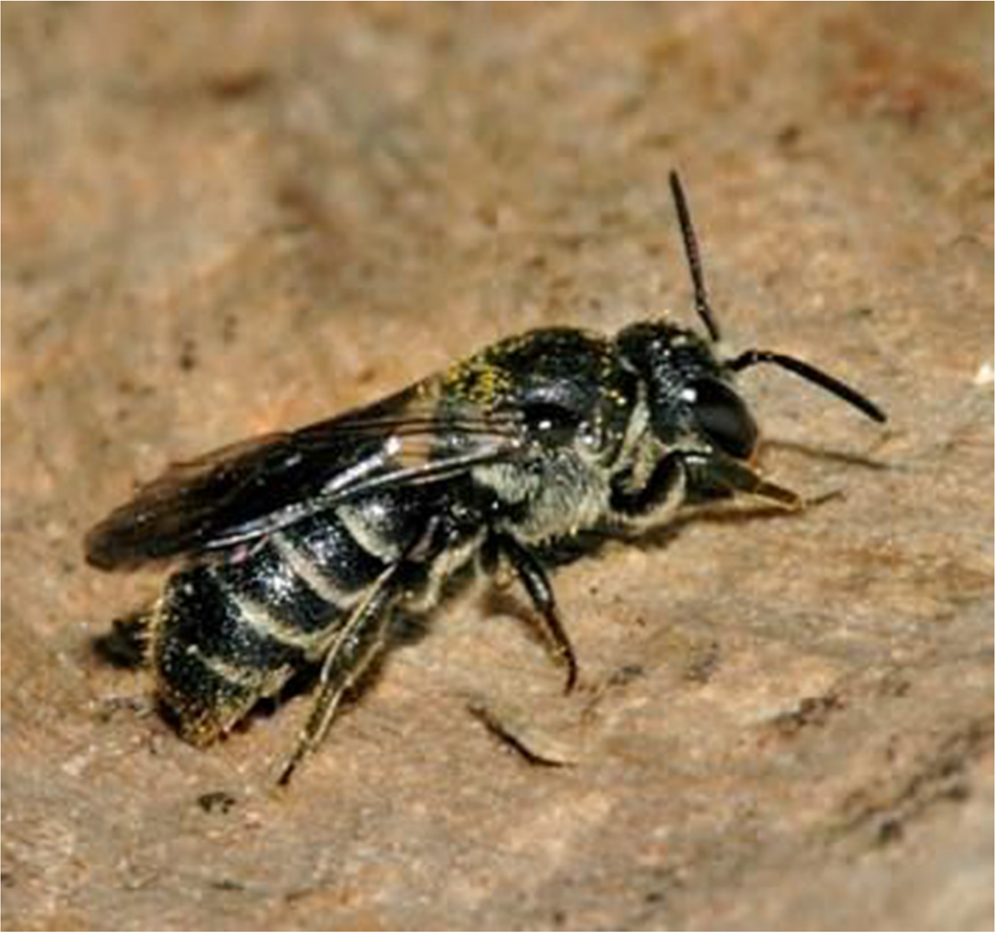
A parasitic bee from the genus *Stelis*.

Turner also was attracted by parasitic bees of the Stelidae family (Figure 3). These bees parasitize the nests of Megachilidae bees, among others. They enter the nests of their hosts and lay their own eggs close to the food supply stored for the host larvae. In this way, their parasitic larvae will consume the food and eventually kill and eat the developing larval host. Yet, Turner was not interested by the parasitism itself but by the orientation of the stelid bees and their response to light and other environmental stimuli (Turner 1911b). He was moved by the question of whether these little bees were responding to light as simple automatic machines, or in fact, exhibited a more complex behavior revealing a sophisticated interplay between various orienting strategies.

He obtained some mud cells in which imago bees were developing and raised the adults within a flight cage in which he studied the copulation and flight behavior. He determined that as most bees, stelids were attracted by sunlight when leaving the nest but he argued that phototaxis (attraction to light) provides an incomplete description of the complexity of behavior of these bees. He showed that parasitic bees use multiple strategies in addition to sunlight orientation. When sun cues were not available, the bees used backup strategies like the position of the nest relative to landmarks, so that they were efficiently oriented. He thus refused to describe the bees’ behavior as that of a machine driven by automatic responses towards environmental cues. The conclusion of his article on the way and extent to which the stelid bees were responding to light, illustrates clearly this position. He wrote therein: “…it seems to the present writer that the reaction of these bees towards the light resembles more the response of a small boy to the music of a brass band than it does the turning of a magnetic needle towards the pole.”

## Turner’s works on honey bees: did he discover their color vision?

An important and repeated claim (Abramson 2003; Bailey 2020) concerning Turner’s work is that he may have discovered honey bee color vision, which would be attributed incorrectly to the Austrian physiologist and Nobel-Prize winner Karl von Frisch. Turner published a series of experiments on the capacity of bees to see colors in 1910 (Turner 1910), while von Frisch’s classical paper on this topic was published 4 years later (Frisch 1914). Before this publication, von Frisch advertised his findings in short communications (e.g., Frisch 1913) but without providing a precise account of his experiments, which were described in detail for the first time in 1914 (Frisch 1914).

**Figure 4.**
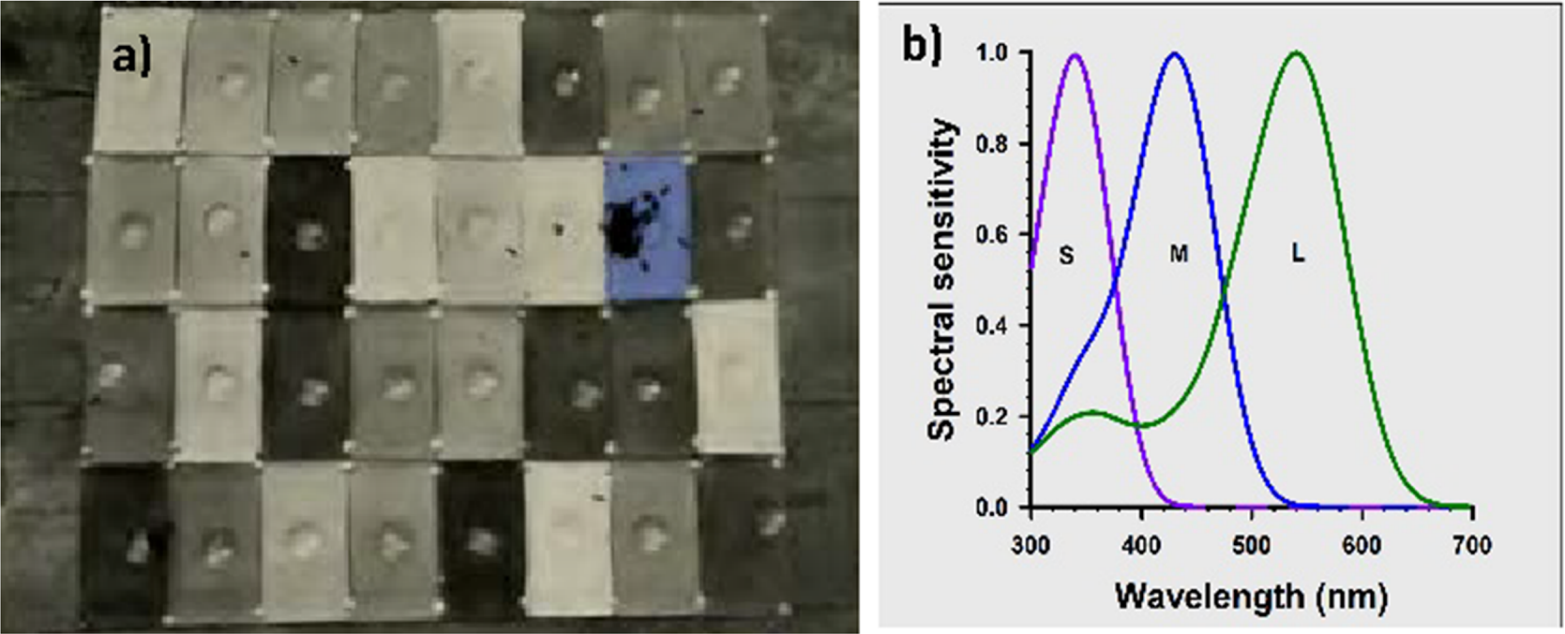
**a** Karl von Frisch’s basic experimental design to demonstrate color vision in honey bees (from [19]). Bees were trained to collect sucrose solution on a dish placed on a blue cardboard. Bees chose the trained color and did not confuse it with achromatic alternatives presenting, in some cases, similar intensity. **b** Spectral sensitivity curves of honey bee photoreceptors, peaking in the UV (S photoreceptor), blue (M photoreceptor), and green range of the spectrum (L photoreceptor).

Color vision is defined as the capacity to distinguish colored surfaces based on their different chromatic contents, independently of intensity differences (Wyszecki and Stiles 1982). Before von Frisch, several scientists suggested that bees may see colors (e.g., Lubbock 1883; Forel 1901; Lovell 1910). Yet, none of them provided the precise experimental evidence showing this capacity as in all cases the demonstration that color choice was independent of differences in intensity was absent. Von Frisch, on the contrary, provided this demonstration using achromatic gray cardboards of variable intensity, which he opposed to the specific color cardboards to which his bees were trained (Figure 4a). He showed that bees learned to associate different color cardboards with a reward of sucrose solution, and that in choosing a rewarded color, they distinguished it from different levels of achromatic gray cardboards, some of which displayed an intensity similar to that of the color trained (Figure 4a). He used 16 colored cardboards varying from violet to red and purple (as seen by humans). This method proved that bees could see the majority of his cardboards as colored surfaces, except in the case of red, which was confused with a black cardboard (Frisch 1914). Later, Kühn extended the demonstration of bee color vision to the ultraviolet range using spectral lights produced by a mercury lamp. In this way, it was demonstrated that bees can see and discriminate colors in the range of 300 nm (ultraviolet) to orange-reddish (650 nm) (Kühn 1924).

The physiological basis for this capacity is the presence of three types of spectral photoreceptors in the bee retina, which set the basis for their trichromatic color vision (Daumer 1956). Their sensitivity peaks are located at 344 nm in the short-wave (ultra violet) region of the spectrum (S receptor), 436 nm in the middle-wave (blue) region (M receptor), and 544 nm in the long-wave (green) region of the spectrum (L receptor), respectively (Autrum and Zwehl 1964; Menzel and Blakers 1976) (Figure 4b).

Four years before the appearance of von Frisch’s massive work on honey bee color vision in 1914 (188 pages and 24 figures) (Frisch 1914), Turner published a brief account termed *Experiments on Color Vision of the Honey Bee* (22 pages, 3 drawings) (Turner 1910) where he explicitly addressed the question of whether honey bees are able to see and distinguish colors. He defined this question as “a matter of much theoretical importance for the correct interpretation of the relations of insects to flowers” (Turner 1910). The article summarized 32 brief experiments and observations performed in the field during 6 days (July 12 to 18, 1910).

**Figure 5.**
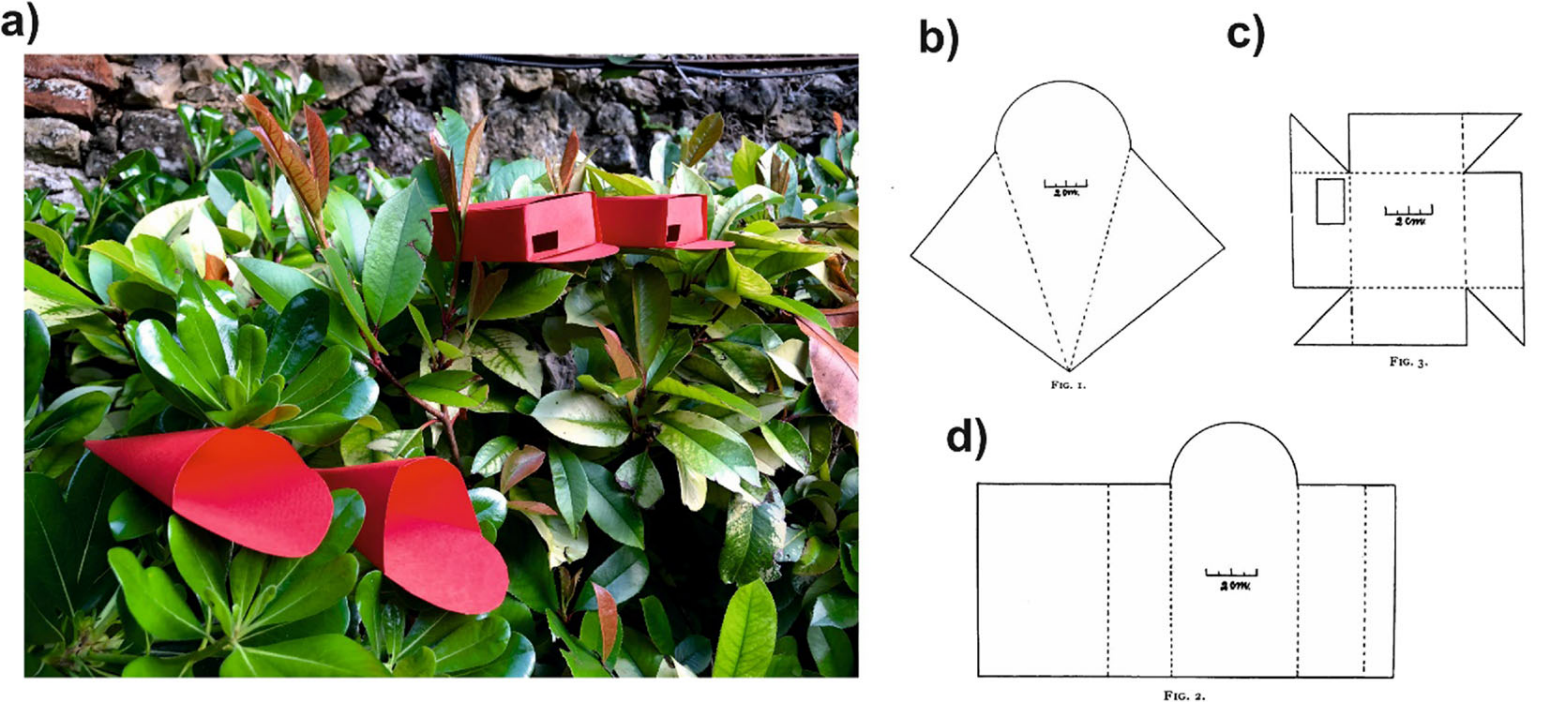
**a** Real-size reconstruction of Turner stimuli (red cones and boxes). Turner placed honey inside them to attract the bees. **b** Original description of a cornucopia provided in Turner’s article. **c** Inner tray and **d** rectangular external case, which defined a box used by Turner in his experiments. Each box had a porch-like extension in front and an open end to allow showing the tray from behind. **b**–**d** from [18]. Thanks to the accurate descriptions provided by Turner in his work, it was possible to reconstruct his stimuli in an exact way 110 years later.

Turner used artificial stimuli (Figure 5), which he placed among blossoms of *Melilotus* sp., where he detected many bees foraging at a time (Turner 1910). In all cases, he baited the stimuli with honey to attract the bees. He performed three series of experiments, varying the type of stimuli used to train the bees: cardboard discs, cardboard cones (or cornucopia), and cardboard boxes with a small opening, which allowed bees to enter to collect the honey (Figure 5).

The stimuli that were rewarded with honey were made unfortunately of red cardboard. Although we do not have the spectral reflection curve of the red he used, the choice of human red made them probably achromatic to the bees. At that time, Turner could not know that bees are blind to red colors. Although there is no question that bees can see such stimuli (Chittka and Waser 1997; Reisenman and Giurfa 2008), and that they can be trained to achromatic (e.g., black) discs and patterns (Giurfa and Menzel 1997), it is probable that in his experiment he was scrutinizing achromatic vision rather than true color vision. In some experiments, he presented blue or green non-rewarded alternatives to prove that bees remained truthful to the red rewarded stimuli but without proper controls it is difficult to prove that bees were choosing to avoid a non-rewarded chromatic (blue, green) stimulus rather than choosing a rewarded achromatic (red) stimulus.

His experiments showed, in any case, that bees used both visual and olfactory cues in their choice behavior. They approached the stimuli attracted by visual cues (red surface, shape), but in the absence of honey odor and, eventually, scent marks (Free 1987), they rejected them, until their enhanced appetitive motivation moved them to accept it. This shows that in most of Turner’s experiments, not only visual cues but also olfactory ones were determinant.

To sum up, Turner did not demonstrate color vision in bees before von Frisch. The choice of red as the rewarded color in all his experiments was unfortunate, but the most important point is the absence of demonstration, available in von Frisch’s work, that visual-stimulus choice was unaffected by variations in the achromatic dimension of stimulus intensity. Had he opposed his red stimuli to black (or dark gray) ones, he may have discovered—as von Frisch did (Frisch 1914)—that bees confused them, and thus that what he was observing was not a case of true color vision. Interestingly, Turner was aware of this problem; he explicitly wrote, when discussing his findings, “whether this is a true color vision or simply a greyness discrimination is no easy question to answer.” Yet, he preferred to conclude that his findings revealed true color vision based on the observation that bees preferred the red stimuli both under the sunshine and under the shadow. Clearly, he knew that it was necessary to control this aspect but he did not perform such a control, probably because the experiments were done under naturalistic conditions and during a very short period.

There are, nevertheless, other merits in Turner’s work, which refer to the way in which he conceived the behavior of the bees. He explained the choice of his artificial stimuli in terms of “meaning acquisition” and even stated that “those things [the stimuli] had acquired a meaning; those strange red things had come to mean ‘honey bearers’, and those strange green things and strange blue things had come to mean ‘not-honey bearers’.” This account reminds the Pavlovian notion of conditioned stimuli (CS) being associated with unconditioned stimuli (US) (Pavlov 1927) and with the principle of stimulus substitution stating that the CS acquires the value of the original US as a result of conditioning (García-Hoz 2014). In this way, Turner anticipated fundamental principles of associative-learning theories. Such elaboration was absent in von Frisch’s work.

## Turner’s experiments on visual pattern discrimination by honey bees

After publishing his experiments on color vision, Turner performed a different series of experiments on honey bees, this time on pattern vision (Turner 1911a). He used cornucopias and boxes similar to those shown in Figure 5 with the difference that this time they were covered by axial (horizontal) or longitudinal (vertical) stripes, red and green or black and white, or were mottled red and green. In this way, he aimed at answering the question of whether bees can discriminate between different patterns. He performed 19 experiments, in which he varied the kind and number of stimuli displayed and showed that bees learned to choose the stimuli, which presented the pattern that had been previously associated with honey. Confronting vertical vs horizontal striped patterns yielded no doubts: despite showing the same colors (e.g., green and red) and the same spatial frequency (i.e., same stripe spacing), bees preferred the previously rewarded vertical-stripe pattern and ignored the horizontal-stripe pattern.

Turner concluded that bees learn and recognize color patterns and that in doing so they learn the spatial distribution of colors (e.g., a vertical and a horizontal grating are not the same). The use of the red color in his red and green patterns indicates again that for bees, the patterns integrated chromatic (green) and achromatic (red) cues. Yet, irrespective of this caveat, the demonstration that bees discriminated previously rewarded patterns from patterns containing the same information but arranged differently remains valid. Here again, he insisted on the notion of “meaning acquisition” by a stimulus and wrote “Lack of response to a stimulus does not mean that that stimulus has not been noticed by the insects; but that, to them, it has not yet acquired a meaning.” Moreover, opposing dominant views suggesting that bees and other insects should be color blind due to their “primitive nature” (Hess 1913), Turner concluded his article on bee pattern vision with a remarkable evolutionary perspective: “Evidently bees can distinguish between color-patterns, and this is of value to them in recognizing plants that yield honey. Hence, since insects can distinguish colors and the fine details of color pattern, there is nothing about the visual powers of bees that militates against the theory that the colors and the color markings of flowers are adaptations to insect visitors” (Turner 1911a)*.*

## Conclusion: Turner’s legacy in a time for change

Several decades after the publication of Turner’s works on bees, many of his ideas have reappeared in modern accounts of insect cognition without scientists being necessarily aware of his contributions (Dona and Chittka 2020; Giurfa and de Brito Sanchez 2020). Turner’s analyses of honey bee foraging behavior and orientation anticipated important notions of Pavlovian learning and modern theories of insect navigation as well as current views interpreting insect behavior from an associative-learning perspective (Giurfa and Menzel 1997; Giurfa 2003; Chittka and Niven 2009). Turner refused to see bees and other insects as simple reflex machines driven by spontaneous reactions to environmental stimuli. For him, behind the insects’ decisions, there was learning, memory, and individual variability. His cognitive perspectives on animal behavior, infrequent at his time and scientific environment dominated by behaviorist views, underline his unicity and talent, and how advanced he was to his time.

Recognition of C.H. Turner should go beyond his experimental work and publications, as what impresses in him is the dedication devoted to his many investigations in an environment that was definitely adverse for his creativity and productivity as a scientist. Turner’s times were times in which eugenic theories were used to justify white supremacy, leading to sterilization of many African American women during medical procedures without consent (Lombardo 2011). The fundamental question that will remain unanswered is which accomplishments he had achieved if he had been given the same opportunities that white scientists had in his time. The same question should be raised today when evaluating the possibilities of minorities in academia and, more generally in the society.

Turner should be seen as an inspiration for scientists fighting against different types of social adversity and prejudices (Abramson 2009; Dona and Chittka 2020; Lee 2020). Bee lovers and beekeepers should learn to discover and appreciate the work of this admirable scientist who made fundamental discoveries and remained nevertheless ignored during decades. We have now a unique opportunity to achieve reparation, recognize, and reward “invisible” black scientists like him and, through this, identify existing injustice in our society for which urgent changes are needed.

## Data Availability

N.A.
